# Binding of Superantigen Toxins into the CD28 Homodimer Interface Is Essential for Induction of Cytokine Genes That Mediate Lethal Shock

**DOI:** 10.1371/journal.pbio.1001149

**Published:** 2011-09-13

**Authors:** Gila Arad, Revital Levy, Iris Nasie, Dalia Hillman, Ziv Rotfogel, Uri Barash, Emmanuelle Supper, Tomer Shpilka, Adi Minis, Raymond Kaempfer

**Affiliations:** Department of Biochemistry and Molecular Biology, Institute of Medical Research Israel-Canada, Hebrew University-Hadassah Medical School, Jerusalem, Israel; National Jewish Medical and Research Center/Howard Hughes Medical Institute, United States of America

## Abstract

Bacterial superantigens, a diverse family of toxins, induce an inflammatory cytokine storm that can lead to lethal shock. CD28 is a homodimer expressed on T cells that functions as the principal costimulatory ligand in the immune response through an interaction with its B7 coligands, yet we show here that to elicit inflammatory cytokine gene expression and toxicity, superantigens must bind directly into the dimer interface of CD28. Preventing access of the superantigen to CD28 suffices to block its lethality. Mice were protected from lethal superantigen challenge by short peptide mimetics of the CD28 dimer interface and by peptides selected to compete with the superantigen for its binding site in CD28. Superantigens use a conserved β-strand/hinge/α-helix domain of hitherto unknown function to engage CD28. Mutation of this superantigen domain abolished inflammatory cytokine gene induction and lethality. Structural analysis showed that when a superantigen binds to the T cell receptor on the T cell and major histocompatibility class II molecule on the antigen-presenting cell, CD28 can be accommodated readily as third superantigen receptor in the quaternary complex, with the CD28 dimer interface oriented towards the β-strand/hinge/α-helix domain in the superantigen. Our findings identify the CD28 homodimer interface as a critical receptor target for superantigens. The novel role of CD28 as receptor for a class of microbial pathogens, the superantigen toxins, broadens the scope of pathogen recognition mechanisms.

## Abstract 2

Bacterial superantigen toxins bind directly to the dimer interface of CD28, the principal co-stimulatory receptor, to induce a lethal cytokine storm, and peptides that prevent this binding can suppress superantigen lethality.

## Author Summary

Induction of protective immunity involves the expression of inflammatory cytokines, proteins that mediate and respond to immune signals. However, excessive cytokine induction can lead to disease, including, at very high levels, lethal toxic shock. Staphylococcal and streptococcal superantigens are a broad family of bacterial protein toxins that induce such a lethal cytokine storm, orders of magnitude higher in intensity than that elicited during normal immune responses. A key participant in every immune response is the costimulatory receptor CD28, which forms a protein dimer to mediate the immune response. Hitherto, CD28 was not known to bind microbial components. Here, we show that superantigens co-opt CD28 as their receptor and that to induce a cytokine storm, superantigens must bind directly into the dimer interface of CD28. The interaction between CD28 and the bacterial superantigen can be blocked with peptides—short protein fragments that mimic the contact domains in the intact superantigen or in CD28. These peptides attenuate inflammatory cytokine gene induction and thus protect animals from lethal toxic shock. Our finding that CD28 is a receptor for the superantigen toxins broadens the scope of microbial pathogen recognition mechanisms and provides a novel approach for designing therapeutics that protect against toxic shock.

## Introduction

As the principal costimulatory receptor, CD28 is a critical regulator of the immune response [1]–[3]. Expressed constitutively on T cells, CD28 is a homodimer that interacts with its B7 coligands to transduce the signal essential for an immediate T cell response [2]–[5]. B7-2 is expressed constitutively and induced immediately, whereas B7-1 is expressed only later [5],[6]; hence, interaction of B7-2 with CD28 regulates signaling by antigens early in the immune response [7],[8]. Apart from its B7 coreceptors, CD28 has no other known ligands.

Bacterial superantigens are a family of proteins secreted by *Staphylococcus aureus* and *Streptococcus pyogenes* that induce toxic shock by activating an immune response, orders of magnitude higher in intensity than that elicited by regular antigens. Bypassing restricted presentation of conventional antigens which typically activate <0.01% of T cells, superantigens bind directly as intact proteins to most major histocompatibility class II (MHC-II) and T cell receptor (TCR) molecules, activating up to 20% of T cells [9]–[11]. This results in massive induction of the T helper 1 (Th1) cytokines interleukin-2 (IL2), interferon-γ (IFN-γ), and tumor necrosis factor (TNF) that mediate toxic shock. Nevertheless, superantigen action also requires CD28 costimulation [12]–[14].

Induction of human T helper 1 (Th1) cytokine gene expression by divergent superantigens is inhibited by a dodecamer peptide that protects mice from their lethal effect [15]. The peptide shows homology to the β-strand(8)/hinge/α-helix(4) domain in staphylococcal enterotoxin (SE) B, structurally conserved in superantigens yet remote from MHC-II and TCR binding sites. Because this domain is essential for superantigen action [15], we considered that it may engage a third receptor. We show here that this receptor is CD28. This costimulatory molecule is co-opted by superantigens as receptor. Superantigens must bind directly into the homodimer interface of CD28 to elicit toxicity. Blocking access of a superantigen to CD28 is sufficient to block lethal toxic shock. Peptide mimetics of the CD28 dimer interface inhibited the induction of Th1 cytokines and protected mice from lethal superantigen exposure. These experiments identify the CD28 homodimer interface as a receptor target for superantigen toxins and reveal a novel role for CD28 in pathogen recognition.

## Results

### Superantigen Mimetic Peptide Inhibits SEB Action and CD28 Signaling

**Figure 1:**
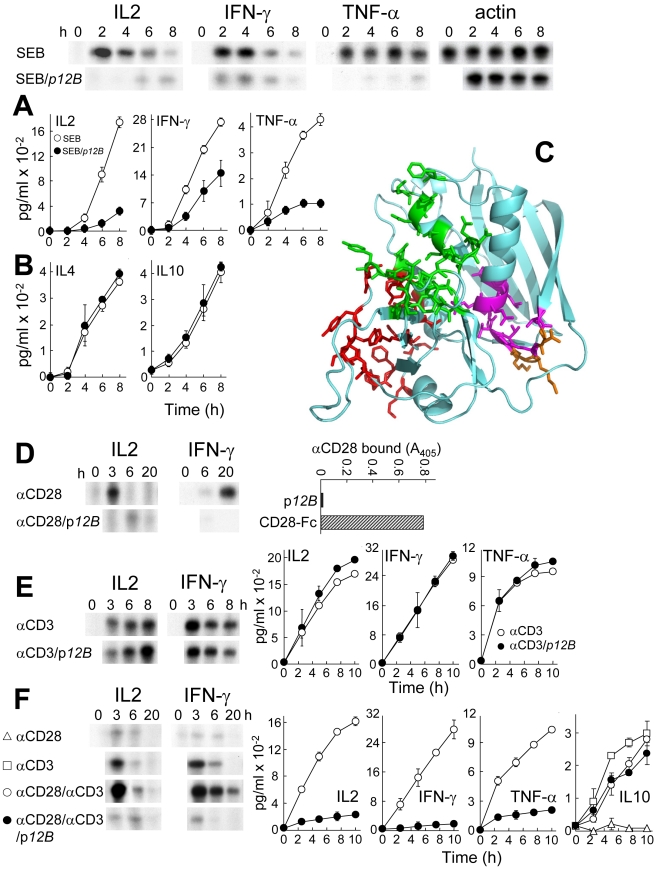
Superantigen mimetic peptide blocks SEB action and CD28 signaling. (A, B) p*12B* (10 µg/ml) inhibits SEB-mediated induction in human PBMC of *IL2*, *IFN-γ*, and *TNF-α* mRNA (autoradiograms) and of protein (graphs; data are shown as means ± SEM (*n* = 3 experiments)) (A) but not of IL4 and IL10 (B). mRNA was quantitated by RNase protection analysis; *β-actin* mRNA indicates equal loading of RNA. (C) In SEB structure (pdb3seb.ent), amino acid residues in the 150–161 β-strand(8)/hinge/α-helix(4) domain are shown in purple, with solvent-accessible residues [36] N151, K153, and K154 in orange. Residues contacting MHC-II are colored red; those contacting the TCR are colored green [37]. (D) p*12B* inhibits induction of *IL2* and *IFN-γ* mRNA in PBMC by αCD28 mAb. PBMC were induced by αCD28 (2.5 µg/ml) alone or with 10 µg/ml p*12B*. To show that αCD28 does not bind p*12B*, p*12B* and CD28-Fc were immobilized and binding of mouse αCD28 was assayed. (E) p*12B* fails to inhibit induction of *IL2*, *IFN-γ*, and *TNF-α* genes by αCD3. PBMC were incubated with αCD3 (0.1 µg/ml) alone or with 10 µg/ml p*12B*. mRNA and secreted cytokines were quantitated. (F) p*12B* inhibits αCD28/αCD3-mediated induction of *IL2*, *IFN-γ*, and *TNF-α* genes but not of IL10. PBMC were incubated with αCD3, αCD28, or both, with or without 10 µg/ml p*12B*. mRNA and secreted cytokines were determined.

Induction of cytokine genes in human PBMC by superantigens and their lethality in mice is blocked by YNKKKATVQELD (p*12A*), a peptide variant of SEB_150–161_, TNKKKVTAQELD, the β-strand(8)/hinge/α-helix(4) domain that is conserved among divergent superantigens [15]. The more stable peptide VQYNKKKATVQELD (p*12B*) carrying VQ from SEB_148–149_ inhibited SEB-mediated induction of *IL2*, *IFN-γ*, and *TNF-α* mRNA and protein (Figure 1A) but not induction of Th2-type cytokines IL4 and IL10 (Figure 1B). Thus, the balance of cytokines induced by SEB was shifted towards the Th2 type, which can explain why mice protected from lethal superantigen challenge by YNKKKATVQELD swiftly acquired immunity against further challenges and developed broadly protective immunoglobulins [15],[16].

Mimetic peptide p*12B* acts early during the immediate reponse to the superantigen, blocking mRNA induction within hours, with inhibition evident already by 2 h (Figure 1A), showing the sensitivity of the mRNA determination. Protein levels reflect this inhibition at the later times, by 6–8 h. These results demonstrate an inhibition of de novo gene induction.

Binding sites in SEB for TCR and MHC-II are remote from the β-strand(8)/hinge/α-helix(4) domain (Figure 1C). We postulated that Th1 cytokine induction requires an additional receptor and that a superantigen binds this receptor via its accessible β-strand(8)/hinge/α-helix(4) domain, rendering this binding sensitive to competition by peptide mimetics.

CD28-deficient mice are resistant to superantigen toxicity and this was explained by the need for B7/CD28 costimulation [13],[14]. SEB-mediated induction of *IL2* and *IFN-γ* genes in human PBMC was inhibited by αB7-2 but not by αB7-1 monoclonal antibody (mAb); by contrast, induction of IL4 and IL10 was resistant (Figure S1A). Moreover, *IL2* and *IFN-γ* mRNA induction by SEB was inhibited selectively by soluble CD28 comprising its extracellular domain fused to IgG1-Fc dimer (CD28-Fc) (Figure S1B). Although CD28-Fc could inhibit SEB action by competing for B7-2, we considered that it might compete with cell-surface CD28 for SEB. Because induction of Th1 but not Th2 cytokines relies on B7-2/CD28 signaling and is selectively inhibited by p*12B*, we hypothesized that superantigens use CD28 as receptor.

To study the role of CD28 directly, we induced *IL2* and *IFN-γ* mRNA with an activating mAb, αCD28. This induction was blocked by p*12B* (Figure 1D). αCD28 failed to bind p*12B*, suggesting that p*12B* competes with αCD28 in binding to cell-surface CD28. *IL2*, *IFN-γ*, and *TNF-α* gene induction by αCD3 was resistant to p*12B* (Figure 1E), showing that p*12B* does not block signaling through the TCR. By contrast, induction of Th1 cytokine gene expression by αCD3 jointly with αCD28, a model for the normal immune response [17], was severely inhibited by p*12B* (Figure 1F). Thus, the peptide blocks a Th1 cytokine response transduced through CD28. p*12B* did not inhibit IL10 induction, implying that it leaves signaling through the TCR intact. αCD28 failed to induce IL10 or to enhance its induction by αCD3. These results show that as in dendritic cells [18], IL10 induction in PBMC is independent of CD28. This explains the observed resistance of SEB-mediated IL10 induction to p*12B*, αB7-2, and CD28-Fc. Because a peptide mimetic of the β-strand(8)/hinge/α-helix(4) domain blocked CD28-mediated Th1 cytokine induction even in the absence of SEB, we considered that superantigens may engage CD28 through this domain.

### Functional Interaction between Superantigen and CD28

**Figure 2:**
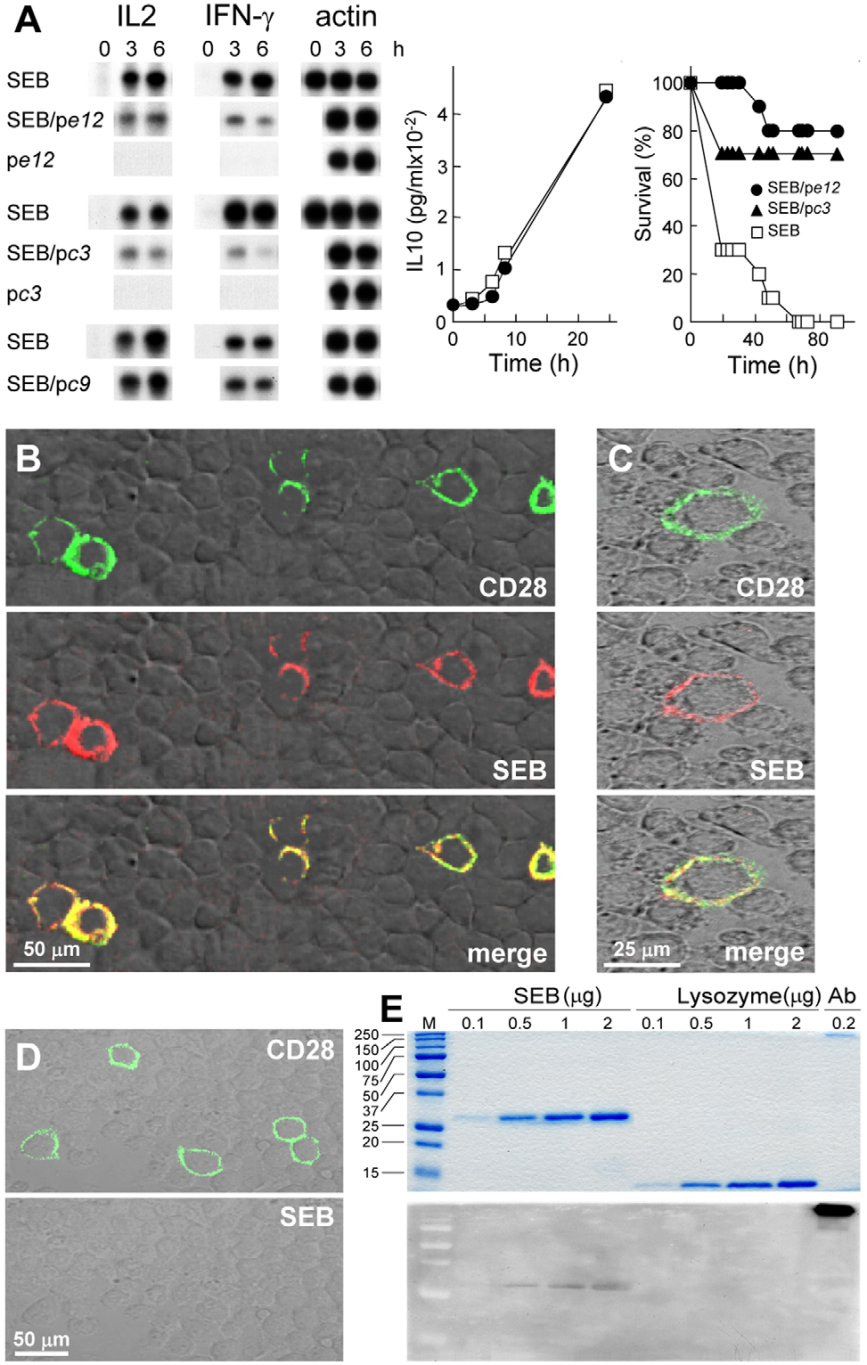
SEB binds to CD28. (A) Phage display peptides selected by affinity for the SEB binding site in CD28 are SEB antagonists that protect mice from killing by SEB. PBMC were induced with SEB alone or with 0.1 µg/ml p*c3*, p*e12*, or p*c9*, a negative control. *IL2* and *IFN-γ* mRNA are shown; *β-actin* mRNA indicates equal loading of RNA. For p*e12*, IL10 was determined (data are shown as means ± SEM (*n* = 3 experiments)). Mice (*n* = 10 per group) were challenged with 6 µg SEB alone or with 0.2 µg p*e12* or 0.5 µg p*c3*; *p* for survival, 10^−4^. (B–D) Binding of SEB to cell surface CD28. Representative fields of confocal microscopy are shown. In (B), HEK293-T cells were transfected to express CD28-GFP fusion protein (green) and after 48 h incubated for 1 h with Alexa-Fluor-633-labeled SEB (red). In (C), BHK-21 hamster cells were transfected with *CD28* cDNA vector and after 48 h incubated successively for 30 min with labeled SEB (red), goat polyclonal αCD28, and Cy2-labeled donkey anti-goat IgG (green). In (D), BHK-21 cells were transfected to express CD28 and after 48 h incubated first with goat polyclonal αCD28 and Cy2-labeled donkey anti-goat IgG (top) and only then with labeled SEB (bottom). (E) Binding of SEB to CD28. SEB, lysozyme, and polyclonal αCD28 (Ab) were separated on duplicate SDS-PAGE gels. Coomassie blue staining (top); far-western blot with CD28-Fc (bottom); M, size marker.

To obtain evidence for a direct, functional interaction between superantigen and CD28, we repeatedly panned a random 12-mer phage-display library on immobilized CD28-Fc, displacing bound phages with SEB. We sequenced 36 peptides and analyzed three for SEB antagonist activity. Phage display peptides p*e12* (SHFTHNRHGHST) and p*c3* (FHKHKNPGSPII) but not the related p*c9* (FHKHNYKSPPII) inhibited *IL2* and *IFN-γ* mRNA induction by SEB but not induction of IL10, shown for p*e12* (Figure 2A). In about equimolar ratio to SEB, p*e12* and p*c3* protected 8/10 and 7/10 mice from lethal challenge that left no survivors in the control group. Thus, superantigen antagonists effective in vivo were selected from random peptide sequences solely by affinity for the SEB binding site in CD28.

### SEB Binds to Cell-Surface CD28

We next expressed human CD28 having GFP fused C-terminally to its intracellular domain in transfected cells (Figure 2B). SEB bound to the surface of cells that expressed CD28 but not to cells in the same field that failed to express CD28. Independently, expression of natural human CD28 and binding of SEB colocalized to the cell surface (Figure 2C). Binding of anti-CD28 Ab to CD28-expressing cells blocked subsequent binding of labeled SEB (Figure 2D). Thus, SEB binds to cell-surface CD28 even in the absence of MHC-II or TCR.

### SEB Uses Its β-strand(8)/hinge/α-helix(4) Domain to Bind CD28

**Figure 3:**
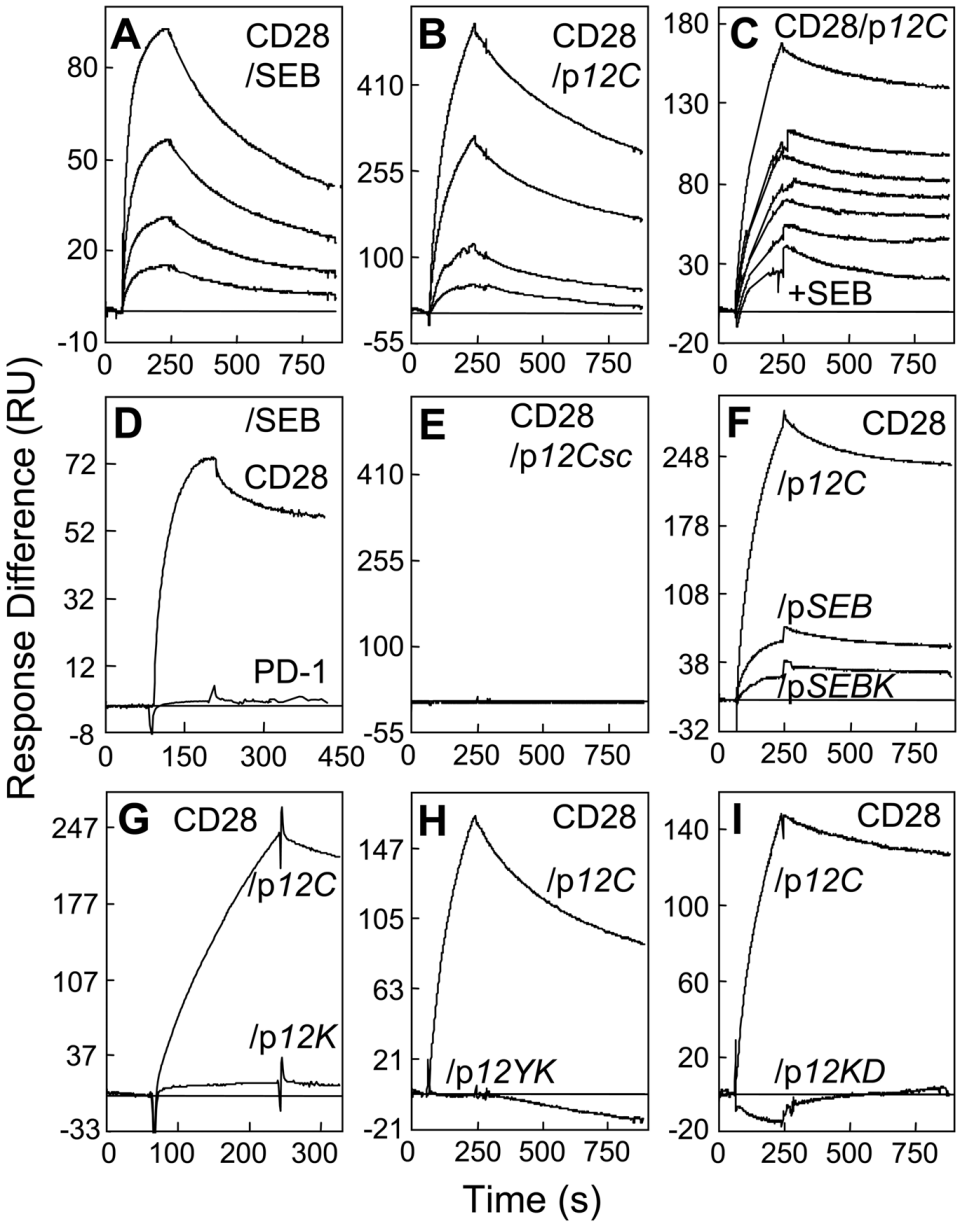
SEB binds CD28 through its β-strand(8)/hinge/α-helix(4) domain. (A and B) Binding of CD28-Fc in 2-fold increments from 0.25 µM to immobilized SEB (A) and from 0.125 µM to p*12C* (B). (C) Binding of CD28-Fc (2 µM) to p*12C* without SEB (top curve) or with SEB in 2-fold increments from 0.78 µM (6 lower curves, top to bottom). (D) Binding of 2 µM PD1-Fc and CD28-Fc to immobilized SEB. (E) Binding of CD28-Fc in 2-fold increments from 0.125 µM to scrambled p*12Csc* (EKAKYTQLVKDN). (F–I) Mutations in SEB β-strand(8)/hinge/α-helix(4) domain peptides reduce binding of 2 µM CD28-Fc. SPR responses are compared for immobilized p*12C* and p*SEB* (TNKKKVTAQELD), p*SEBK* (TNAKKVTAQELD) (F), p*12K* (YNAKKATVQELD) (G), p*12YK* (ANAKKATVQELD) (H), and p*12KD* (YNAKKATVQELA) (I). Representative SPR responses are shown.

SEB bound CD28-Fc directly in far-western analysis whereas lysozyme did not (Figure 2E), showing that binding of SEB to CD28 will survive denaturation and is robust. Using surface plasmon resonance (SPR), we detected binding of purified CD28-Fc but not of human IgG to immobilized SEB (Figures 3A, S2A and S2B).

We next immobilized β-strand(8)/hinge/α-helix(4) domain peptide p*12C* (YNKKKATVQELD abutted by Cys at both termini) which was as active as p*12A* (YNKKKATVQELD abutted by D-Ala at both termini) in inhibiting SEB-mediated *IL2* and *IFN-γ* mRNA induction (Figure S3). CD28-Fc bound p*12C* directly (Figure 3B) and specifically, as no binding was detected for IgG or ubiquitin (Figure S2C and S2D). SEB competitively inhibited binding of CD28-Fc to immobilized p*12C* (Figure 3C). The signal of CD28-Fc binding declined progressively with increasing SEB concentration showing that SEB did not bind to the immobilized peptide. Half-maximal inhibition occurred when SEB was in 1.5-fold excess over CD28-Fc. The finding that SEB free in solution inhibited binding of CD28 free in solution to immobilized superantigen mimetic peptide implies that the two proteins bind each other in solution, independent of any surface consideration and provides direct evidence that SEB uses its β-strand(8)/hinge/α-helix(4) domain to bind CD28. This competition analysis yielded K_D_ = 2.3 µM for complex formation between free SEB and CD28-Fc. Thus, SEB binds CD28 with similar affinity as TCR and MHC-II [19],[20]. By contrast, soluble costimulatory receptor Programmed Death-1 (PD-1) failed to bind SEB (Figure 3D).

Binding of CD28 to the peptide was sensitive to mutations. Random scrambling of p*12C* abrogated CD28 binding (Figure 3E). CD28-Fc bound significantly better to p*12C* than to its natural homolog p*SEB* (Figure 3F); p*SEB* is far weaker as SEB antagonist [15], showing a correlation between the ability of the peptide to bind CD28 and its biological activity as superantigen antagonist. A single alanine substitution in p*SEB* (p*SEBK*) reduced binding by >50% (Figure 3F). A similar substitution in p*12C* (p*12K*) severely reduced binding; pairwise substitutions abolished it (p*12YK*, p*12KD*) (Figure 3G–I). The mutations and scrambling created negative control surface, showing specificity of the binding. Thus, SEB binds CD28 directly through its β-strand(8)/hinge/α-helix(4) domain, explaining the inhibitory effect of mimetic p*12B* on SEB-induced and CD28-mediated Th1 cytokine gene expression.

### CD28 Uses Its Homodimer Interface to Bind SEB

**Figure 4:**
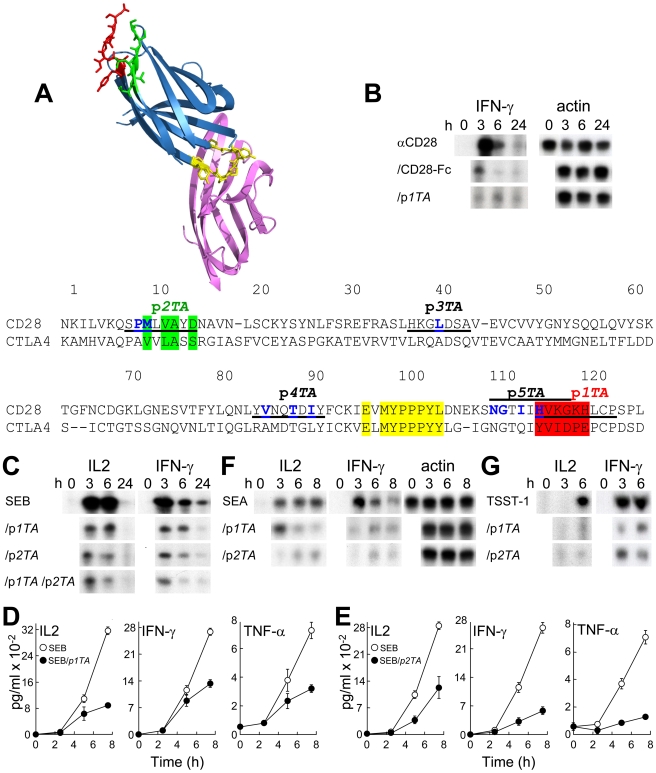
Peptide mimetics of the CD28 dimer interface are superantigen antagonists. (A) CTLA-4/B7-2 complex (1I85.pdb; [1]) and the dimer interface in CTLA-4. B7-2 is shown in purple and CTLA-4 in blue, with MYPPPY in yellow, YVIDPE (HVKGKH in CD28) in red, and VVLASS (MLVAYD in CD28) in green, as in the sequence alignment of the extracellular domains of human CD28 and CTLA-4. Amino acids at the crystallographic dimer interface of CD28 [21] are shown in blue. Positions of peptides are underlined. (B) p*1TA* antagonizes induction of *IFN-γ* mRNA by αCD28. PBMC were induced by αCD28 (2.5 µg/ml) alone or with 10 µg/ml of CD28-Fc or p*1TA*. *IFN-γ* and *β-actin* mRNA was determined. (C–E) CD28 dimer interface peptides p*1TA* and p*2TA* (0.1 µg/ml) inhibit induction by SEB of *IL2* and *IFN-γ* mRNA (C) and IL2, IFN-γ, and TNF-α (D and E) in PBMC (data are shown as means ± SEM (*n* = 3 experiments)). (F and G) Induction of *IL2* and *IFN-γ* mRNA by SEA (F) and TSST-1 (G) alone or with 0.1 (F) or 10 µg/ml peptide (G); *β-actin* mRNA indicates equal loading of RNA.

Since p*12C* bound CD28, the observation that p*12B* blocked signaling by αCD28 suggested that p*12B* competes with αCD28 for its epitope in CD28. Epitope mapping of αCD28 defined CD28_116–124_, HVKGKHLCP; HVKGKH aligns with YVIDPE in CTLA-4 (Figure 4A) [1]. CD28 and CTLA-4 are homologous and fold similarly [21] yet their sequences differ in this domain and in residues 10–15, likely to prevent heterodimer formation [1],[5]. In the folded CTLA-4 protein, these two domains are juxtaposed, creating the dimer interface. The conserved B7 binding domain MYPPPY is located on the opposite side. Peptide HVKGKHLCP (p*1TA*) abrogated induction of IFN-*γ* mRNA by αCD28 (Figure 4B), functional evidence that αCD28 indeed engages CD28_116–124_, part of the putative CD28 dimer interface [21]. p*1TA* inhibited cytokine gene induction by SEB (Figure 4C and 4D), implying that to elicit a Th1 response, SEB must bind CD28 at a site overlapping with HVKGKHLCP.

We next synthesized SPMLVAYD (p*2TA*; CD28_8–15_), a mimetic of the second CD28 dimer interface domain predicted from alignment with CTLA-4 (Figure 4A). In the available structure for CD28, p*2TA* overlaps with the dimer interface; most of the flexible p*1TA* domain was not resolved [21]. Like p*1TA*, p*2TA* antagonized SEB-mediated induction of Th1 cytokine genes (Figure 4C and 4E). The p*1TA*/p*2TA* combination was not more potent (Figure 4C). These results provide strong evidence that the functional superantigen binding site in CD28 is composite and includes dimer interface residues in p*1TA* and p*2TA*.

**Figure 5:**
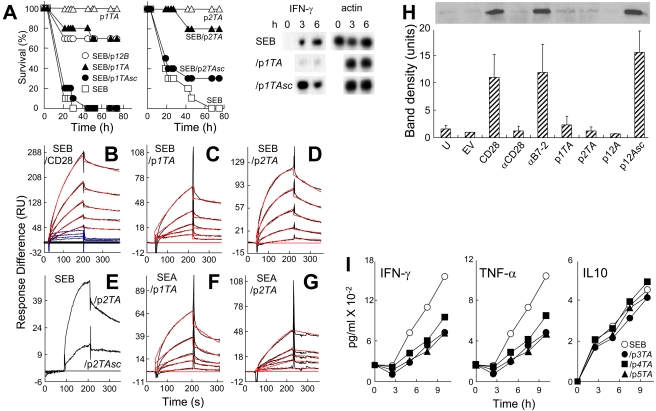
CD28 dimer interface mimetic peptides bind and antagonize superantigens and protect mice from lethal challenge. (A) Mice (*n* = 10 per group) were challenged with 6 µg SEB alone or with 1 µg p*1TA*, 5 µg p*12B*, or 1 µg scrambled peptide p*1TAsc* (CHGHLVPKK), or with 0.2 µg p*2TA* or p*2TAsc* (ASMDYPVL); controls without SEB received 25 µg peptide; *p* for survival, 2×10^−8^ (p*1TA*), 0.38 (p*1TAsc*), 3×10^−6^ (p*2TA*), and 0.19 (p*2TAsc*). PBMC were induced with SEB alone or with 1 µg/ml p*1TA* or p*1TAsc*; *IFN-γ* and *β-actin* mRNA was determined. (B–G) Representative SPR responses for binding of SEB to immobilized CD28-Fc, p*1TA*, and p*2TA* (B–D), of SEB to p*2TAsc* or *p2TA* (E) and of SEA to p*1TA* and p*2TA* (F and G). Graphical fits to the binding curves are presented in red color; kinetic parameters show the specificity of these interactions (Table S1). Analyte concentrations increased in 2-fold increments from 0.156 (C and F), 0.312 (G), 1.56 (B), and 3.12 µM (D); in (E), binding of p*2TA* and p*2TAsc* is shown for the highest of 5 concentrations tested, 10 µM. In (B), curves in blue color show binding of ribonuclease A in four 2-fold increments from 3.12 µM to immobilized CD28-Fc. (H) Peptide mimetics of the CD28 dimer interface and of the β-strand(8)/hinge/α-helix(4) domain in SEB inhibit binding of SEB to cell-surface CD28. 293-T cells were transfected to express CD28 and after 36 h incubated for 1 h without addition (CD28) or with 0.1 µg/ml αCD28, anti-B7-2 or 10 µg/ml p*1TA*, p*2TA*, p*12A*, or its scrambled form p*12Asc* (EKAKYTQLVKDN), before further incubation for 1 h with 15 µg/ml *wt* SEB. Cells were washed 3 times with cold phosphate-buffered saline before lysis. Western blot of equal amounts of total cell protein (Bradford assay) with αSEB mAb and horseradish peroxidase-conjugated goat anti-mouse IgG shown is from a representative experiment. Bar graph shows intensity of bands for three independent experiments ± SD. U, untransfected; EV, empty vector. (I) IFN-γ, TNF-α, and IL10 (data are shown as means ± SEM (*n* = 3)) in medium from PBMC induced with SEB alone or with 0.1 µg/ml p*3TA*, p*4TA*, or p*5TA*.

SEB engages only the MHC-II α-chain whereas SEA binds also the β-chain, increasing its affinity for MHC-II over 10-fold [22]–[24]. SEA was as sensitive as SEB to p*1TA* and p*2TA* (Figure 4F). Thus, interaction with the CD28 dimer interface is used generally by superantigens and not merely to compensate for weak binding of SEB to MHC-II. Even though the superantigen toxic shock syndrome toxin-1 (TSST-1) is only 6% homologous with SEB and binds the TCR via a different domain [25], the β-strand(8)/hinge/α-helix(4) domains in TSST-1 and SEB show spatial conservation [15]. Indeed, p*1TA* and p*2TA* also inhibited IL2 and IFN-*γ* mRNA induction by TSST-1 (Figure 4G).

p*1TA* and p*2TA* blocked superantigen lethality. Whereas no controls (0/10) survived SEB challenge, 7/10 mice survived that received p*1TA* shortly before SEB exposure (Figure 5A). p*1TA* was protective when present in 3.6-fold excess over SEB. Moreover, in only 0.8-fold ratio to SEB, p*2TA* protected 8/10 mice from lethal shock. Scrambling p*1TA* sequence abolished its ability to block IFN-*γ* mRNA induction by SEB. Randomly scrambled peptides p*1TAsc* and p*2TAsc* failed to provide significant protection of mice from the lethal effect of SEB, showing specificity for p*1TA* and p*2TA*. p*12B* in 12-fold excess over SEB gave comparable protection. We conclude that the CD28 dimer interface plays a critical role in mediating the deleterious response to a superantigen.

To validate the concept that the superantigen binding site in CD28 is the dimer interface, we next examined whether p*1TA* and p*2TA* bind superantigens directly. To overcome limitations of SPR affinity measurement using divalent ligands, we analyzed binding of SEB to immobilized CD28-Fc (Figure 5B) which yielded K_D_ = 2.3 µM under conditions where the highest analyte concentrations approached 10-fold the K_D_, allowing for an accurate estimation; this K_D_ agrees well with that derived from competition analysis (Figure 3C). Immobilized CD28-Fc readily bound B7-2-Fc (Figure S2E) but not ribonuclease (Figure 5B), a control protein similar in size to SEB, supporting specificity of SEB binding. SEB bound to immobilized p*1TA* and p*2TA* (Figure 5C and 5D), with K_D_ values in the micromolar range (Table S1). Binding to p*2TAsc* was severely impaired (Figure 5E), showing specificity for the natural CD28 sequence. Likewise, SEA bound p*1TA* and p*2TA* (Figure 5F and 5G). Short peptide mimetics of the CD28 dimer interface, p*1TA* and p*2TA*, bind superantigens directly and exhibit broad-spectrum superantigen antagonist activity, apparently because they compete with cell-surface CD28 for the β-strand(8)/hinge/α-helix(4) domain in the superantigen.

We used western blotting to show that p*1TA*, p*2TA*, and p*12A* but not scrambled p*12Asc* block binding of SEB to cells expressing CD28 (Figure 5H). SEB binding was abrogated by αCD28 but insensitive to anti-B7-2 Ab. Thus, two classes of peptides block the interaction between superantigen and CD28: mimetics of the CD28 homodimer interface and of the β-strand(8)/hinge/α-helix(4) domain in superantigens.

CD28 peptides p*3TA*, p*4TA*, and p*5TA* harbor additional contact residues in the crystallographic dimer interface of CD28 (Figure 4A). These mimetic peptides inhibited IFN-γ and TNF-α induction to the same extent as p*1TA* and p*2TA* but had no effect on IL10 induction (Figure 5I). Thus, each of five peptides covering distinct portions of the CD28 dimer interface (Movie S1, Figure S4) is a superantigen antagonist.

SEB induced a transient change in surface accessibility of epitope HVKGKHLCP at the CD28 homodimer interface (Figure S5). The epitope was not detected by αCD28 at 0 and 24 h, showing that it is shielded in the dimer, but became accessible 6 h after exposure to SEB. Most plausibly, by opening the dimer interface and engaging one CD28 monomer, SEB renders the second monomer accessible to αCD28.

### Toxicity of SEB Is Abrogated by Mutation of Its CD28-Binding Domain

**Figure 6:**
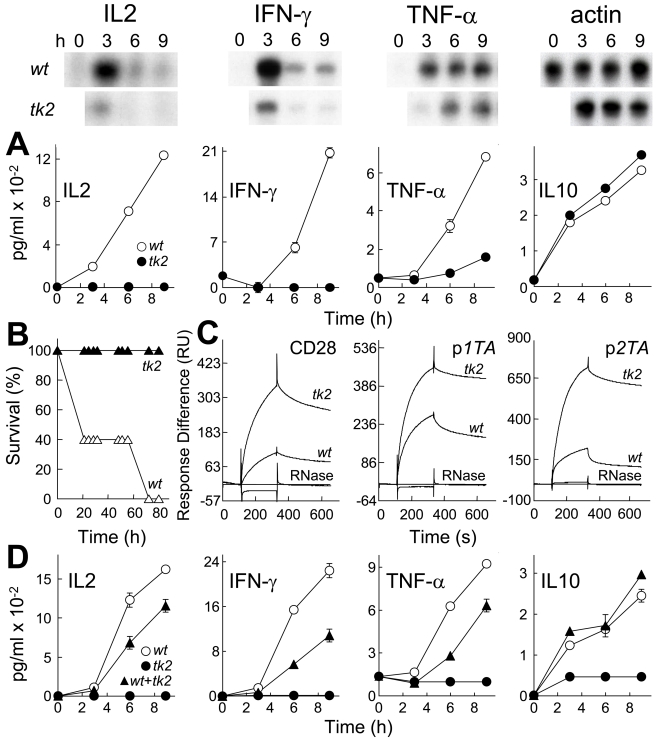
Mutation of SEB β-strand(8)/hinge/α-helix(4) domain affects binding to CD28, Th1 cytokine gene induction and lethality. (A) Induction of cytokine genes by *wt* and *tk2* mutant SEB. PBMC were induced with 1 ng/ml *wt* or *tk2* SEB; mRNA (autoradiograms) and secreted cytokines (graphs; data are shown as means ± SEM (*n* = 3)) are depicted. (B) Lack of lethality of *tk2* SEB. Mice (*n* = 5 per group) were challenged with 10 µg *wt* or *tk2* SEB; *p* for survival, 10^−5^. (C) Affinity of *tk2* SEB for CD28. Representative SPR responses for binding of *wt* and *tk2* SEB to immobilized CD28-Fc, p*1TA*, and p*2TA* are shown for 5 µM *wt* and *tk2* SEB or RNase A to facilitate comparison; kinetic data were collected for five 2-fold increments in protein concentration from 1.25 µM (Table S2). (D) Dominant-negative phenotype of *tk2* SEB. PBMC were induced with 1 ng/ml *wt* SEB, 0.1 ng/ml *tk2* SEB, or both; secreted cytokines were determined as shown (data are shown as means ± SEM (*n* = 3)).

Experiments with peptides capable of blocking Th1 cytokine induction in human PBMC and lethal shock in mice show that SEB must bind CD28 to exert its toxicity. We next mutated the β-strand(8)/hinge/α-helix(4) domain in holo-SEB. SEB*^T150A/K152A^* (*tk2*) carries alanine substitutions in two highly conserved residues [15] critical for binding of CD28 in peptide context (Figure 3G and 3H) and adjacent to solvent-exposed residues (Figure 1C). The *tk2* mutation severely impaired the ability of SEB to induce *IL2*, *IFN-γ*, and *TNF-α* mRNA and protein yet left induction of IL10 unabated (Figure 6A), evidence that the ability to signal through MHC-II and TCR remained fully intact, an essential control showing that the protein was not denatured by the mutation. The *tk2* mutation abolished the lethality of SEB. No mice (0/5) survived challenge with wild type (*wt*) SEB yet all (5/5) survived challenge with *tk2* (Figure 6B). Thus, an intact β-strand(8)/hinge/α-helix(4) domain is essential for SEB toxicity.

The *tk2* mutation enhanced binding of SEB to CD28-Fc, maximal response increasing 3-fold (Figure 6C); whereas *wt* SEB again yielded a K_D_ in the micromolar range, for *tk2* the affinity reached nanomolar values (Table S2). This result was unexpected given the loss of lethality of *tk2* and lack of affinity of p*12YK* for CD28-Fc. Enhanced binding of *tk2* did not result from interaction with a different site in CD28, as it was accompanied by increased binding to both p*1TA* and p*2TA*. Loss of the lethality of *tk2* and of its ability to induce Th1 cytokines shows that the interaction of *tk2* with CD28 is nonproductive. We considered that the higher affinity of *tk2* for CD28 might render it dominant-negative. Indeed, even when present at a 10-fold lower concentration, *tk2* significantly reduced the induction of IL2, IFN-*γ*, and TNF-α by *wt* SEB (Figure 6D). Yet *tk2* induced IL10 additively with *wt* SEB, confirming the selectivity of the dominant-negative phenotype. The *tk2* mutation, which affects affinity for CD28 but not signaling through MHC-II/TCR, shows that direct engagement of CD28 by the superantigen via its β-strand(8)/hinge/α-helix(4) domain is critical for a deleterious Th1 cytokine response. To elicit productive signaling for Th1 cytokine gene induction and lethality, the superantigen must bind CD28 with moderate affinity.

**Figure 7:**
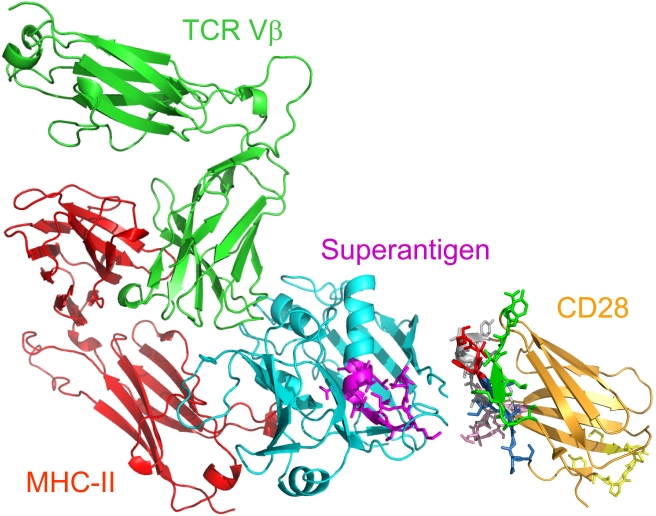
Model for superantigen action through binding to CD28. Structural model for Th1 cell activation by superantigens through simultaneous binding to CD28, MHC-II, and TCR. SEC3 (cyan), a close relative of SEB, in complex with TCR Vβ (green) and MHC-II (red) extracellular domains (1jck.pdb; [38]). The SEC3 β-strand/hinge/α-helix domain in magenta is freely accessible to the N-terminal 118-residue portion of the CD28 extracellular domain (1yjd.pdb; [21]). CD28 homodimer interface peptides (Figure 4A) are shown: p*1TA* (HVK resolved in 1yjd.pdb is red), p*2TA* (green), p*3TA* (pink), p*4TA* (grey), and p*5TA* (SNGTII is blue); MYPPPY is yellow. CD28 and TCR are oriented such that their trans-membrane domains (not shown) can enter the T cell at top; the MHC-II is oriented towards the antigen-presenting cell at bottom; for simplicity, cell surfaces are not shown.

Structural analysis shows that a superantigen can bind simultaneously to CD28 and the TCR on the T cell and MHC-II on the antigen-presenting cell (Figure 7). CD28 can be accommodated readily as third superantigen receptor in the quaternary complex, with the CD28 dimer interface oriented towards the β-strand/hinge/α-helix domain in the superantigen.

## Discussion

These experiments identify the CD28 dimer interface as a receptor target for superantigens. We show that to deliver the signal for Th1 cytokine induction, a superantigen must co-opt CD28 as third receptor, in addition to its full reliance on costimulatory signaling through B7-2/CD28. Thus, superantigens make unconventional use not only of MHC-II and TCR through direct binding but also of CD28. Independent lines of evidence show that the binding site for superantigens in CD28 is the crystallographic homodimer interface. The superantigen domain that engages CD28 contains the conserved β-strand/hinge/α-helix motif [15], remote from MHC-II and TCR binding sites, leaving it accessible to CD28 (Figure 7). Blocking access of a superantigen to CD28 is sufficient to block lethal toxic shock. Mice were protected from lethal SEB challenge by peptide mimetics of the CD28 dimer interface and of the β-strand/hinge/α-helix domain in superantigens, as well as by random peptides selected to compete with SEB for its binding site in CD28. The finding that CD28 is a direct sensor of a class of microbial pathogens, superantigen toxins, broadens the scope of pathogen recognition mechanisms.

Independent lines of evidence support direct binding of superantigen to CD28. SEB bound to cell-surface CD28 even in the absence of MHC-II or TCR. Novel peptide antagonists of SEB, protective against lethal toxic shock, were selected by phage display solely for their affinity for the SEB binding site in CD28. Surface plasmon resonance showed that CD28 binds directly to SEB, whether one or the other was immobilized and even when both were free in solution, and specifically to peptide mimetics of the SEB β-strand(8)/hinge/α-helix(4) domain but not, or poorly, to mutant peptides in which 1–2 amino acids were changed to alanine. Conversely, another mutation in such a mimetic peptide, leading to higher SEB antagonist activity [15], enhanced its affinity for CD28. This peptide blocked binding of SEB to cell-surface CD28. Thus, SEB uses its β-strand(8)/hinge/α-helix(4) domain to bind CD28.

We provide evidence that the superantigen binding site in CD28 is the dimer interface. CD28 mimetic peptides p*1TA* and p*2TA* corresponding to dimer interface sequences each inhibited Th1 cytokine gene induction by superantigens as widely different as SEB, SEA, and TSST-1 and protected mice from lethal SEB challenge even when present in about equimolar ratio to toxin. p*1TA* and p*2TA* directly bound SEB as well as SEA, with affinities close to that of CD28. The antagonist activity of these peptides is explained by their ability to compete with cell-surface CD28 for superantigen. p*1TA* and p*2TA* each blocked binding of SEB to cell-surface CD28. Each of five peptides overlapping distinct portions of the CD28 dimer interface was a superantigen antagonist. We did not perform mutagenesis of the CD28 dimer interface because mutations therein abrogate cell surface expression of the CD28 receptor [21], rendering interpretation problematic.

The CD28 dimer interface has no known role in costimulation and is remote from the B7 binding site. SEB induces vigorous expression of Th1 and Th2 cytokines but only the Th1 response, here defined by induction of *IL2*, *IFN-γ*, and *TNF-α* genes, depends on CD28 engagement. By attenuating this response, antagonist peptides will reduce the synergy between these cytokines, to allow survival. Our data support the interpretation that the early Th2 cytokine response induced by a superantigen, as opposed to Th2 cell differentiation [26], is independent of CD28 signaling and hence resistant to blocking by the mimetic peptides. It is tempting to link the selective need for CD28 signaling in the superantigen-mediated induction of *IL2*, *IFN-γ*, and *TNF-α* genes, but not of *IL4* and *IL10* genes, to the distinct transcription factor dependence for expression of Th1- versus Th2-type cytokine genes, involving, respectively, T-bet [27],[28] and GATA-3 [29].

CD28 monomers do not dimerize at low concentrations, indicating weak noncovalent interaction [21], facilitating displacement by superantigen. Engagement of a superantigen should displace contacts between the CD28 monomers which are linked through a disulfide bond [21] beyond the HVKGKH motif in the dimer interface. This epitope, buried in resting T cells, was rendered accessible upon exposure to SEB. Our data support the interpretation that the superantigen induces conformational change in CD28 that activates signaling, without interfering with B7-2/CD28 costimulation. More commonly, ligand binding induces receptor homodimerization needed for signaling, as for human growth hormone receptor [30] and erythropoietin receptor [31]. In contrast, the superantigen-binding domain of CD28 overlaps with its homodimer interface.

In our mouse model, an accepted model for the lethality of superantigens [15], most if not all of the mice died within 48 h after a single exposure to SEB but they were protected by peptides. By contrast, *HLA DR3* transgenic mice were far less sensitive to SEB challenge, requiring two toxin exposures 48 h apart before death was induced, and no protection by a β-strand(8)/hinge/α-helix(4) superantigen domain mimetic peptide was observed thereafter [32]. The rapid kinetics of toxin action in our mouse model reflects the rapid course of toxic shock, which was blocked by the peptides despite their limited halflife. In human PBMC, induction of Th1 cytokine gene expression, the immediate response occurring during the first hours after superantigen exposure, was sensitive to peptides. Binding of SEB to PBMC proved insensitive to β-strand(8)/hinge/α-helix(4) domain mimetic peptide [32] as expected, because SEB will also bind to the MHC-II and TCR, which unlike CD28 are not targeted by this peptide.

The phenotype of *tk2* mutant SEB shows that superantigen activity not only requires binding of CD28 via the β-strand(8)/hinge/α-helix(4) domain but that this binding must be with moderate affinity. The mutation led to significantly higher affinity for CD28 but this binding was nonproductive, as reflected by the complete loss of lethality of SEB. Mutations in a holoprotein can change protein conformation and thereby affect ligand binding properties. However, conformational changes in an intact protein are not necessarily reflected in short peptides derived from a limited domain; thus, in peptide p*12YK*, the same mutation led to loss of affinity for CD28. Given the similarly moderate affinity of SEB for CD28, TCR, and MHC-II, we propose that it is the concerted interaction of the superantigen with all three receptors that allows for stable synapse formation resulting in exceptionally strong Th1 cytokine induction and lethality. This affinity limitation will render superantigen action sensitive to regulation and allow peptide mimetics that interfere with binding of superantigen to CD28 to disrupt synapse formation, thus preventing toxic shock. Two classes of peptides block the interaction between superantigen and CD28: β-strand/hinge/α-helix domain mimetics that compete with superantigen for CD28 and CD28 homodimer interface mimetics that compete with CD28 for superantigen.

## Materials and Methods

### Ethics Statement

Experiments involving mice were approved by the institutional animal care and use committee.

### Peptides

Peptides were synthesized using fluoronyl-methoxycarbonyl chemistry, cleaved, and the side chain deprotected with triflouroacetic acid. Peptides were >95% pure by high-pressure liquid chromatography; molecular weight was verified by MALDI-TOF mass spectrometry. Peptides were abutted with D-Ala at both termini for greater protease resistance in biological assays and with Cys for SPR. Scrambled sequences were obtained using a true random number generator (http://www.random.org/).

### Soluble CD28, PD-1, and B7-2

CD28, PD-1, and B7-2 expressed in mouse myeloma NS0 cells (R&D Systems) comprise the extracellular 19–152, 24–167, and 20–239 amino acid domain, respectively, of the mature human ligands fused to C-terminal human IgG1 Fc and are homodimers, disulfide-linked in the Fc domain. Soluble ligands were >95% pure as judged by SDS-PAGE.

### Antibodies

Mouse *γ*1 anti-SEB mAb (MB2B33, Toxin Technology), horseradish peroxidase-conjugated goat anti-mouse IgG (KPL); mouse IgG1 anti-human CD28 mAb (MAB342, clone 37407), mouse IgG1 anti-human CD3 epsilon mAb (clone UCHT1), mouse IgG1 anti-human B7-1/CD80 mAb (clone 37710) and anti-human B7-2/CD86 mAb (clone 37301), goat IgG anti-human CD28 polyclonal Ab (AF-342-PB) and goat IgG anti-human B7-2/CD86 polyclonal Ab (AF-141-NF), from R&D Systems. Binding of αCD28 to immobilized CD28-Fc and p*12B* was assayed in enzyme-linked immunosorbent assays using corresponding alkaline phosphatase-coupled αIgG (Jackson Laboratories).

### Induction of Cytokine Gene Expression

Human PBMC were separated on Ficoll Paque (Amersham), washed twice with 50 ml of RPMI 1640 medium, resuspended at 4×10^6^ cells/ml, and cultured in this medium supplemented with 2% fetal calf serum, 2 mM glutamine, 10 mM MEM nonspecific amino acids, 100 mM Na-pyruvate, 10 mM Hepes pH 7.2, 5×10^−5^ M 2-mercaptoethanol, 100 U/ml penicillin, 100 µg/ml streptomycin, and 5 µg ml^−1^ nystatin. SEB (Lot 1430, Department of Toxinology, U.S. Army Medical Research Institute of Infectious Diseases), SEA, and TSST-1 (Sigma) were added to 100 ng/ml. Secreted cytokines, quantitated in triplicate with Quantikine ELISA kits (R&D Systems), are presented as means ± SEM.

### Ribonuclease Protection Analysis

Total RNA was extracted from aliquots of 3×10^7^ PBMC with Trizol reagent (Invitrogen) and hybridized for 18 h at 42°C with genomic antisense RNA probes transcribed with α-[^32^P]UTP from DNA inserted into pBS (Promega). The 600-nt *IL2* probe, a T7 transcript, is complementary to *IL2* exon 3 and part of intron 3; 117 nt of this RNA are protected by *IL2* mRNA [15]. The 274-nt *IFN-γ* probe, a T3 transcript, is complementary to *IFN-γ* exon 3 and part of intron 3; 183 nt of this RNA are protected by *IFN-γ* mRNA [15],[33]. The 570-nt *TNF-α* probe, a T3 transcript, is complementary to parts of *TNF-α* intron 3 and exon 4; 220 nt of this RNA are protected by *TNF-α* mRNA. Antisense RNA probes for 18S rRNA and *β-actin* mRNA protect 90 and 245 nt, respectively.

### Recombinant Superantigens

Chromosomal DNA from *S. aureus* COL and from an SEA-producing strain was used to clone *wt SEB* and *SEA* genes into pHTT7K [34] and express them in *E. coli* as mature proteins with an N-terminal His_6_-tag. PCR primers 5′-AGCTTGTACGTCAAAAGATAATAAAT-3′ and 5′-AATGCGAAAAAGGTGACTGCTCAAG-3′ were used to create *tk2 SEB*. Inserts were verified by DNA sequencing. Total protein was loaded onto a His•Bind column (Novagen) and eluted stepwise with imidazole. Recombinant proteins recovered after dialysis were >98% pure on SDS-PAGE and >98% homogeneous as monomers upon analytical gel filtration through a 1×30 cm Superdex 75 column calibrated with molecular weight standards (GE Healthcare-Amersham Pharmacia) from which proteins were eluted at a flow rate of 1 ml/min. In PBMC, *wt* SEB and SEA induced IL2, IFN-*γ*, TNF-α, and IL10 at 1 ng/ml, attesting to full activity; *wt* SEB was lethal to mice in the same concentration range as natural SEB.

### Binding of SEB to Cell-Surface CD28

A vector expressing CD28-GFP fusion protein was generated from *CD28* cDNA vector template [35] using KOD polymerase (Novagen) with phosphorylated PCR primers 5′-CTCAGATCTCGCCACCATGCTCAGGCTGCTCTTGGCTCT and 5′-CTCGGATCCGGAGCGATAGGCTGCGAAGTC, deleting the *CD28* termination codon. The PCR product was inserted into pEGFP-N3 DNA (Clontech) that had been digested with BamH1 and BglII. *CD28*-transfected cells were incubated successively for 30 min each with *wt* SEB labeled with Alexa Fluor 633 (Molecular Probes), goat polyclonal αCD28 Ab (AF-342-PB, R&D Systems), and Cy2-labeled donkey anti-goat IgG (Jackson Laboratories), washing after each step.

### Far-Western Analysis

*wt* SEB (28.4 kD), egg white lysozyme (14.3 kD; Sigma), or goat polyclonal αCD28 (R&D Systems) were incubated for 5 min at 95°C and separated in duplicate by 12% SDS-PAGE. One gel was stained with Coomassie blue and the other transferred to a Nitrocellulose Transfer Membrane (Whatman, 0.2 mm) in a Semi Dry Transfer instrument (Schleicher & Schuell). The membrane was blocked with 5% skim milk before incubation for 1 h with 0.5 µg/ml horseradish peroxidase-conjugated CD28-Fc (R&D Systems). Binding of CD28-Fc was detected using ECL-plus (Pharmacia).

### Phage Display

For epitope mapping, the PhD-12 combinatorial phage display library in M13KE (New England Biolabs) was panned on immobilized αCD28 mAb (MAB342) following instructions of the manufacturer; displacement was with 100 µg/ml CD28-Fc. Phages from the fourth panning were immobilized on nitrocellulose membranes. Binding of αCD28 was detected with horseradish peroxidase-linked anti-mouse IgG (Jackson Laboratories) using ECL-plus. Sequences of 19 distinct inserts were aligned with CD28, without gaps. For CD28 affinity selection, the same library was panned on immobilized CD28-Fc; bound phages were displaced with 100 µg/ml SEB (Lot 1430). To screen for binding, phages were immobilized and incubated with 0.5 µg/ml horseradish peroxidase-conjugated CD28-Fc. After 4 rounds of panning, >10% of selected phages tightly bound CD28-Fc.

### Surface Plasmon Resonance Spectroscopy

Proteins and peptides were diluted to 10–200 µg/ml in 10 mM Na acetate pH 4.0 and immobilized on a CM5 sensorchip using amine coupling kit and amine-thiol coupling kit (BIAcore), respectively; amounts immobilized were 500–1,000 RU. Analytes were injected at 20 µl/min in 25 mM HEPES pH 7.4, 150 mM NaCl, 3.4 mM EDTA, and 0.005% surfactant P20 under conditions showing no mass transport limitation. Regeneration was with 50 mM phosphoric acid. Kinetic analyses were performed at 25°C in a BIAcore 3000 instrument, deducting the control flow cell signal from the binding signal. Analyte curves were run in duplicate; representative results are shown. BIAevaluation 3.1 software was used to determine K_D_ in the linear ligand concentration range (1∶1 Langmuir binding). To determine K_D_ for the SEB/CD28-Fc complex from data in Figure 3C, CD28-Fc bound to immobilized p*12C* in the presence of increasing concentrations of SEB was quantitated relative to the dose response curve generated for binding of CD28-Fc to p*12C* in the absence of SEB. Standard error as percent of k_a_ and k_d_ and χ^2^ values well below 10 show the quality of fit between calculated and observed binding values and attest to the purity of the ligands examined. Fitting residuals were within the range of ±2, validating the goodness of fit. CD28-Fc was titrated in BIAcore binding assays for the ability to bind to its cognate immobilized mAb. CD28-Fc was also titrated for its ability to bind to immobilized sB7-2. Human IgG (Jackson Laboratories), ribonuclease A, and His_10_-tagged ubiquitin (R&D Systems) served as controls.

### Mouse Lethality Assay

Female BALB/c mice (10–12 wk; Harlan) were challenged by intraperitoneal injection of SEB and 20 mg *D*-galactosamine to sensitize the animals to superantigens [15]. Antagonist peptides were injected intraperitoneally 30 min before challenge. Controls without SEB received peptide 30 min before injection of *D*-galactosamine. Survival was monitored. Viability remained constant beyond 72 h for as long as monitored, 2 wk.

### Statistical Analysis

Survival curves were analyzed using the Kaplan-Meier method, with the Log-Rank test for comparisons.

### Structure Models

Cartoon models of protein structure were created in PyMol (www.pymol.org).

## Supporting Information

Figure S1 Induction of a Th1 cytokine response by SEB relies on B7-2/CD28 signaling. (A) αB7-2 inhibits SEB-induced expression of *IL2* and *IFN-γ* genes but not of IL4 and IL10. PBMC were induced with SEB alone or with 100 ng/ml αB7-1 or αB7-2 monoclonal antibody. Graphs show *IL2* and *IFN-γ* mRNA determined by quantitative dot-blot hybridization (vertical rows in autoradiogram below each graph show 8-h values) [15] and cytokines secreted into culture medium (data are shown as means ± SEM (*n* = 3)). (B) CD28-Fc inhibits SEB-mediated induction of *IL2* and *IFN-γ* mRNA but not of IL10. PBMC were induced with SEB, 1 µg/ml CD28-Fc, or both. *IL2* and *IFN-γ* mRNA was determined by RNase protection analysis; *β-actin* mRNA indicates equal loading of RNA. IL10 level is shown (data are shown as means ± SEM (*n* = 3)).
(PDF)

Figure S2 Binding of CD28 to SEB and to p*12C* is specific. (A) Purity of CD28-Fc (1 µg) was assessed by 12% SDS-PAGE and staining with Coomassie blue. M, molecular weight marker. (B) Representative SPR responses for binding of human IgG in concentrations ranging from 0.25 µM in 2-fold increments to immobilized SEB (Lot 1430). (C, D) Representative SPR responses for binding of human IgG (C) and His_10_-tagged ubiquitin (D) in concentrations ranging from 0.125 µM in 2-fold increments to immobilized p*12C*. (E) Representative SPR responses for binding of B7-2-Fc in 2-fold increments from 0.03 µM to CD28-Fc immobilized as in Figure 5B.
(PDF)

Figure S3 Mimetic peptide p*12C* is an active SEB antagonist. PBMC were induced with SEB alone or with 100 ng/ml p*12A* or p*12C*. *IL2* and *IFN-γ* mRNA were quantitated by RNase protection analysis (autoradiograms).
(PDF)

Figure S4 Antagonist peptides in the CD28 dimer interface. A still of Movie S1. In CD28 extracellular domain (1yjd.pdb), location is shown of homodimer interface peptides p*1TA* (HVK resolved in 1yjd.pdb is red), p*2TA* (green), p*3TA* (pink), p*4TA* (grey), and p*5TA* (SNGTII is blue) (see Figure 4A); MYPPPY is yellow.
(PDF)

Figure S5 SEB induces a transient change in surface accessibility of CD28 epitope HVKGKHLCP. CD4 cells were enriched to 90% from PBMC by use of RosetteSep (Stem Cell Technologies), incubated at a density of 4×10^6^ cells/ml and before addition of SEB (0 h), or at times indicated, washed, and stained with αCD28 mAb (secondary Ab: Cy-2, green) or goat polyclonal CD28 Ab (secondary Ab: Cy-3, red). Confocal fluorescence microscopy is shown. Merge, double staining with both antibodies.
(PDF)

Movie S1 Antagonist peptides in the CD28 dimer interface. In CD28 extracellular domain (1yjd.pdb), location is shown of homodimer interface peptides p*1TA* (HVK resolved in 1yjd.pdb is red), p*2TA* (green), p*3TA* (pink), p*4TA* (grey), and p*5TA* (SNGTII is blue) (see Figure 4A); MYPPPY is yellow.
(MOV)

Table S1 Kinetic parameters of surface plasmon resonance analysis in Figure 5.
(PDF)

Table S2 Kinetic parameters of surface plasmon resonance analysis in Figure 6.
(PDF)

## Abbreviations

GFP: green fluorescent protein
IFN: interferon
IL: interleukin
MHC-II: major histocompatibility class II
SE: staphylococcal enterotoxin
SPR: surface plasmon resonance
TCR: T cell receptor
Th: T helper
TNF: tumor necrosis factor
TSST-1: toxic shock syndrome toxin-1
wt: wild type
