# Correction: Binding of Superantigen Toxins into the CD28 Homodimer Interface Is Essential for Induction of Cytokine Genes That Mediate Lethal Shock

**DOI:** 10.1371/journal.pbio.1002237

**Published:** 2015-08-21

**Authors:** Gila Arad, Revital Levy, Iris Nasie, Dalia Hillman, Ziv Rotfogel, Uri Barash, Emmanuelle Supper, Tomer Shpilka, Adi Minis, Raymond Kaempfer

The authors would like to clarify that for some of the actin RNA loading control panels in Figure 2A and those in Figure 6A, a temporary placeholder was left in inadvertently. In Figure 2A, actin RNA was not assayed for p*c9*, as this phage display peptide was used as a negative control that failed to inhibit cytokine mRNA induction by SEB. Since equal amounts of RNA were loaded for electrophoretic analysis, the conclusion remains valid. Figure 6A now displays the actual actin RNA panels. tk2 mutant SEB is impaired in its ability to induce cytokine gene expression; reduced cytokine mRNA levels are reflected by reduced protein levels.

Corrected versions of Figs 2 & 6 are included here.

**Fig 2:**
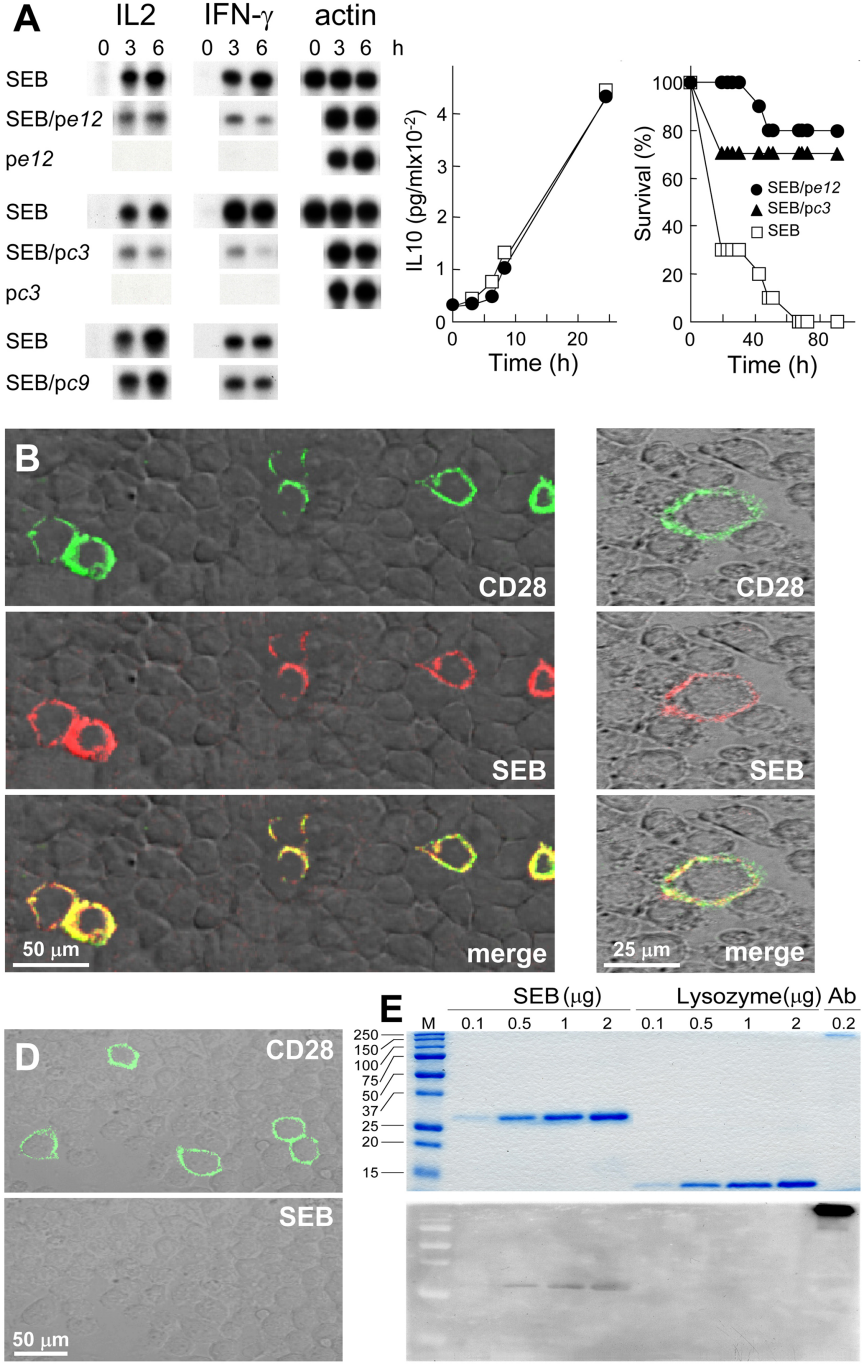
SEB binds to CD28. (A) Phage display peptides selected by affinity for the SEB binding site in CD28 are SEB antagonists that protect mice from killing by SEB. PBMC were induced with SEB alone or with 0.1 μg/ml p*c3*, p*e12*, or p*c9*, a negative control. *IL2* and *IFN-γ* mRNA are shown; *β-actin* mRNA indicates equal loading of RNA. For p*e12*, IL10 was determined (data are shown as means ± SEM (*n* = 3 experiments)). Mice (*n* = 10 per group) were challenged with 6 μg SEB alone or with 0.2 μg p*e12* or 0.5 μg p*c3*; *p* for survival, 10^−4^. (B–D) Binding of SEB to cell surface CD28. Representative fields of confocal microscopy are shown. In (B), HEK293-T cells were transfected to express CD28-GFP fusion protein (green) and after 48 h incubated for 1 h with Alexa-Fluor-633-labeled SEB (red). In (C), BHK-21 hamster cells were transfected with *CD28* cDNA vector and after 48 h incubated successively for 30 min with labeled SEB (red), goat polyclonal αCD28, and Cy2-labeled donkey anti-goat IgG (green). In (D), BHK-21 cells were transfected to express CD28 and after 48 h incubated first with goat polyclonal αCD28 and Cy2-labeled donkey anti-goat IgG (top) and only then with labeled SEB (bottom). (E) Binding of SEB to CD28. SEB, lysozyme, and polyclonal αCD28 (Ab) were separated on duplicate SDS-PAGE gels. Coomassie blue staining (top); far-western blot with CD28-Fc (bottom); M, size marker.

**Fig 6:**
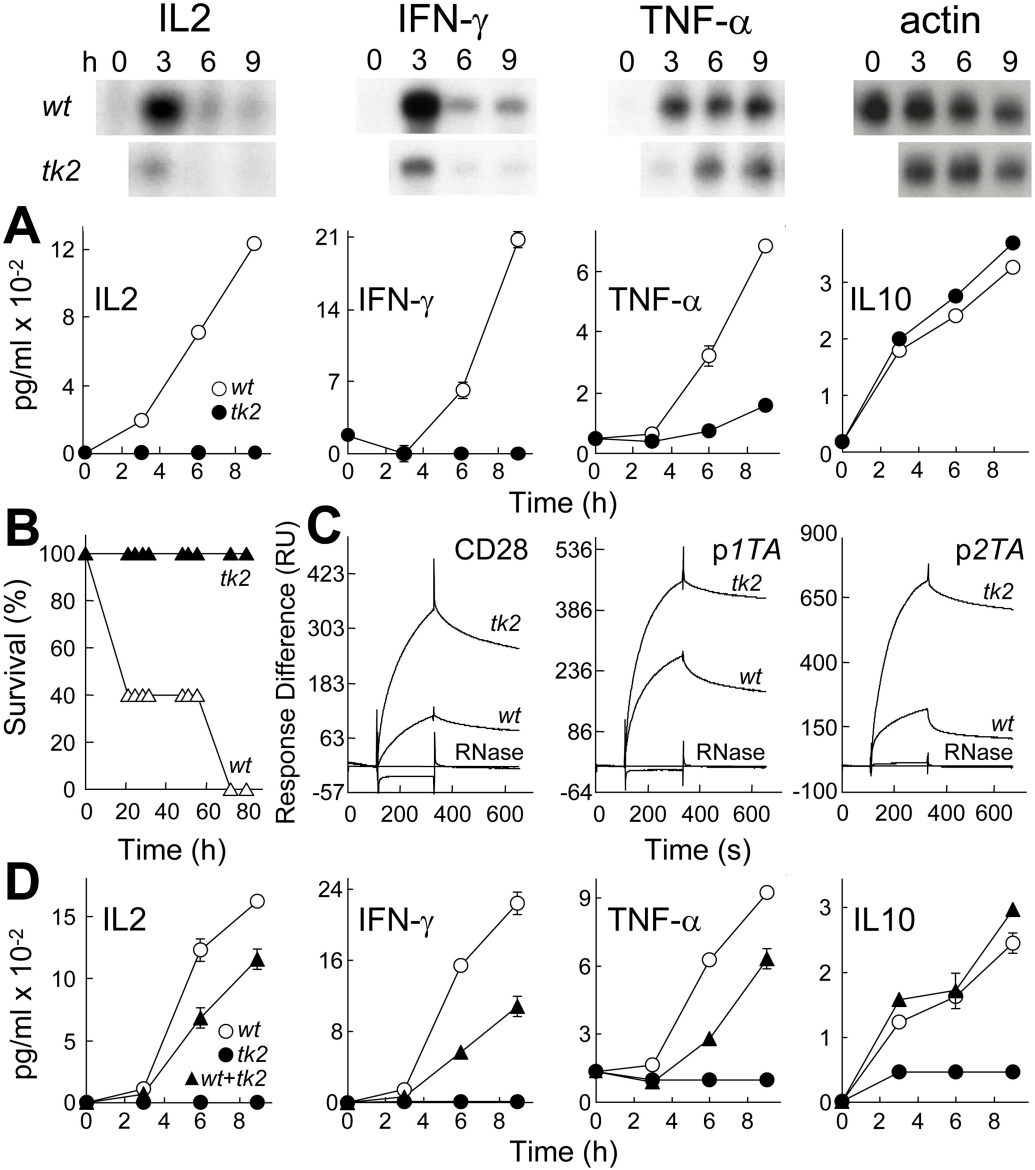
Mutation of SEB β-strand(8)/hinge/α-helix(4) domain affects binding to CD28, Th1 cytokine gene induction and lethality. (A) Induction of cytokine genes by *wt* and *tk2* mutant SEB. PBMC were induced with 1 ng/ml *wt* or *tk2* SEB; mRNA (autoradiograms) and secreted cytokines (graphs; data are shown as means ± SEM (*n* = 3)) are depicted. (B) Lack of lethality of *tk2* SEB. Mice (*n* = 5 per group) were challenged with 10 μg *wt* or *tk2* SEB; *p* for survival, 10^−5^. (C) Affinity of *tk2* SEB for CD28. Representative SPR responses for binding of *wt* and *tk2* SEB to immobilized CD28-Fc, p*1TA*, and p*2TA* are shown for 5 μM *wt* and *tk2* SEB or RNase A to facilitate comparison; kinetic data were collected for five 2-fold increments in protein concentration from 1.25 μM (Table S2). (D) Dominant-negative phenotype of *tk2* SEB. PBMC were induced with 1 ng/ml *wt* SEB, 0.1 ng/ml *tk2* SEB, or both; secreted cytokines were determined as shown (data are shown as means ± SEM (*n* = 3)).
