# Supplementary figures and images for: Binding of Superantigen Toxins into the CD28 Homodimer Interface Is Essential for Induction of Cytokine Genes That Mediate Lethal Shock
Source: PLoS Biol. 2011 Sep 13;9(9):e1001149. doi: 10.1371/journal.pbio.1001149 (PMC3172200; doi:10.1371/journal.pbio.1001149)

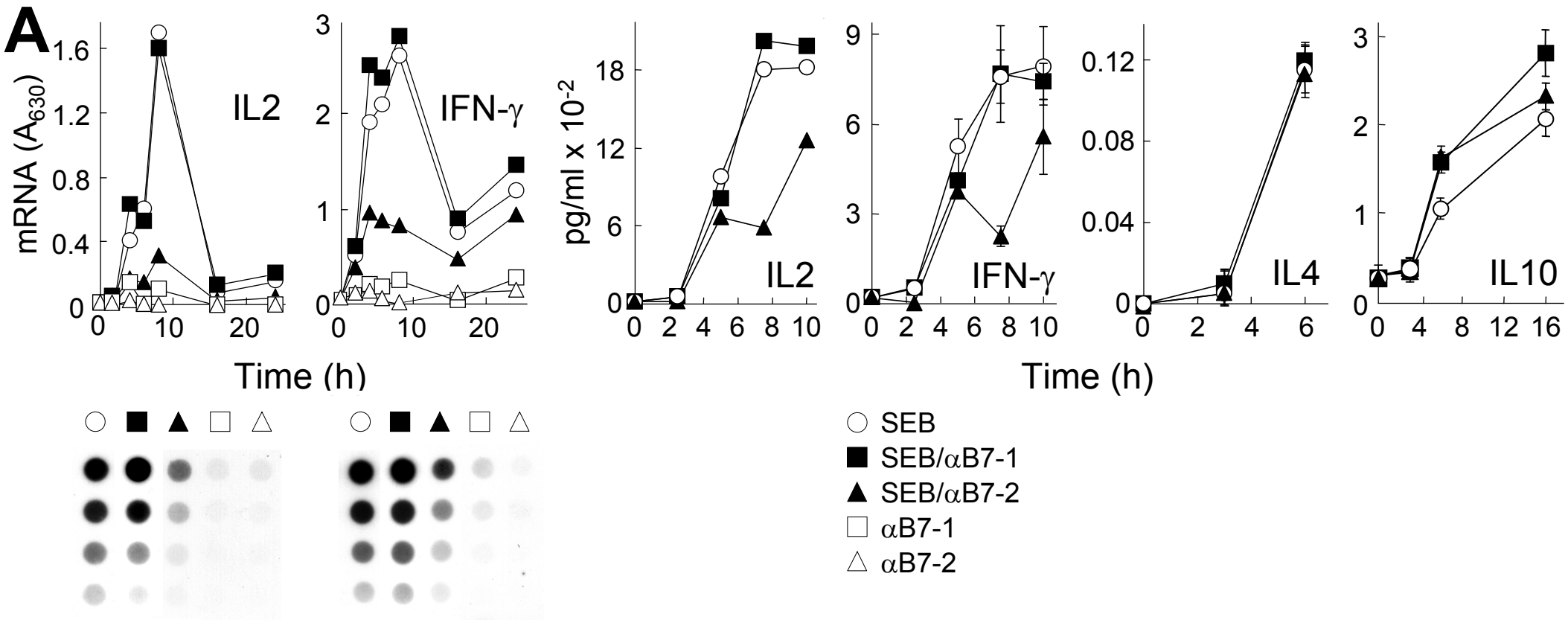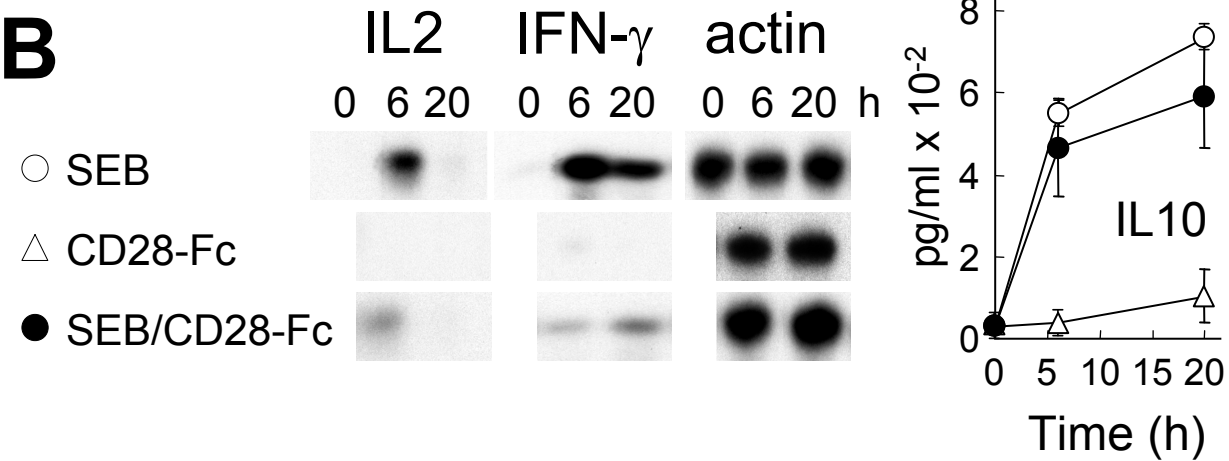

Supplement: Figure S1 — Induction of a Th1 cytokine response by SEB relies on B7-2/CD28 signaling. (A) αB7-2 inhibits SEB-induced expression of IL2 and IFN-γ genes but not of IL4 and IL10. PBMC were induced with SEB alone or with 100 ng/ml αB7-1 or αB7-2 monoclonal antibody. Graphs show IL2 and IFN-γ mRNA determined by quantitative dot-blot hybridization (vertical rows in autoradiogram below each graph show 8-h values) [15] and cytokines secreted into culture medium (data are shown as means ± SEM (n = 3)). (B) CD28-Fc inhibits SEB-mediated induction of IL2 and IFN-γ mRNA but not of IL10. PBMC were induced with SEB, 1 µg/ml CD28-Fc, or both. IL2 and IFN-γ mRNA was determined by RNase protection analysis; β-actin mRNA indicates equal loading of RNA. IL10 level is shown (data are shown as means ± SEM (n = 3)). (PDF) [file pbio.1001149.s001.pdf]

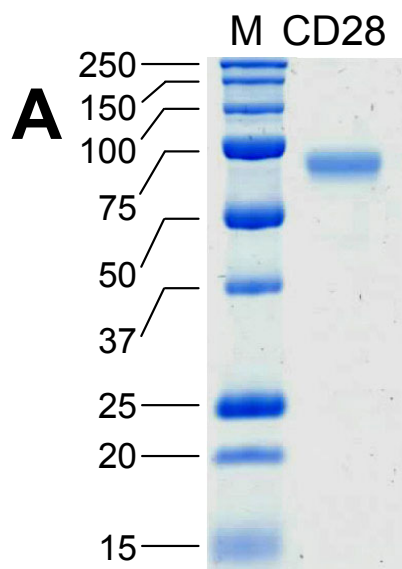

Response Difference (RU)

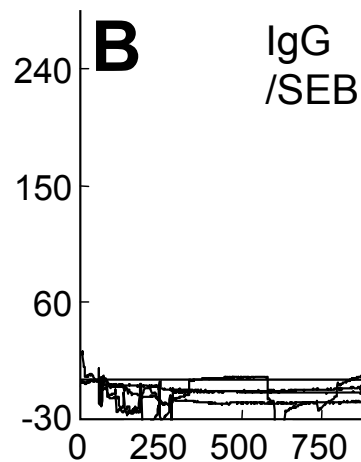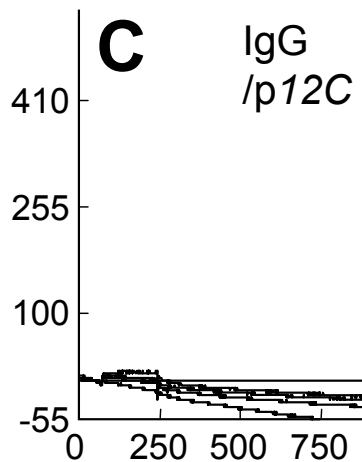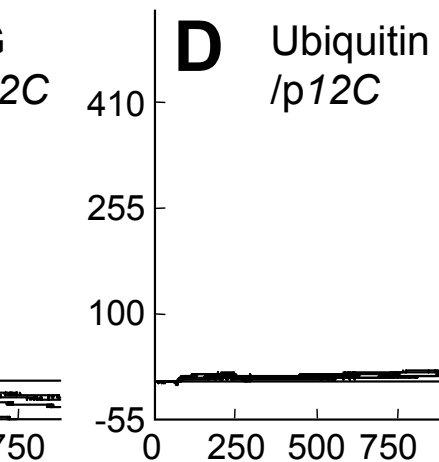

Time (s)

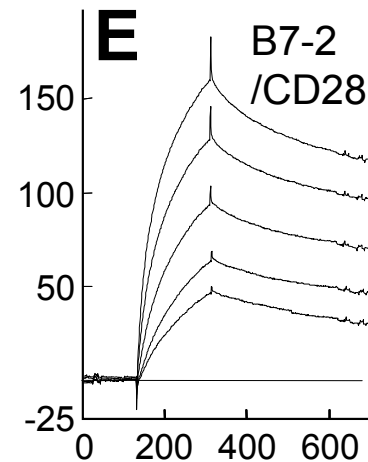

Supplement: Figure S2 — Binding of CD28 to SEB and to p12C is specific. (A) Purity of CD28-Fc (1 µg) was assessed by 12% SDS-PAGE and staining with Coomassie blue. M, molecular weight marker. (B) Representative SPR responses for binding of human IgG in concentrations ranging from 0.25 µM in 2-fold increments to immobilized SEB (Lot 1430). (C, D) Representative SPR responses for binding of human IgG (C) and His10-tagged ubiquitin (D) in concentrations ranging from 0.125 µM in 2-fold increments to immobilized p12C. (E) Representative SPR responses for binding of B7-2-Fc in 2-fold increments from 0.03 µM to CD28-Fc immobilized as in Figure 5B. (PDF) [file pbio.1001149.s002.pdf]

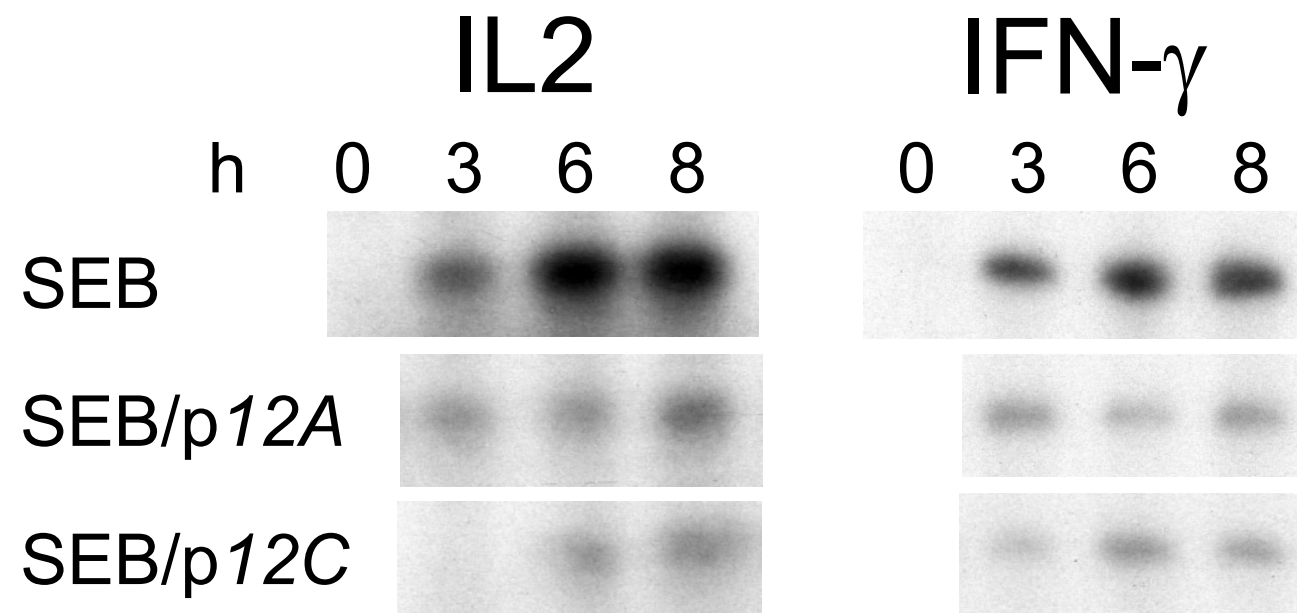

Supplement: Figure S3 — Mimetic peptide p12C is an active SEB antagonist. PBMC were induced with SEB alone or with 100 ng/ml p12A or p12C. IL2 and IFN-γ mRNA were quantitated by RNase protection analysis (autoradiograms). (PDF) [file pbio.1001149.s003.pdf]

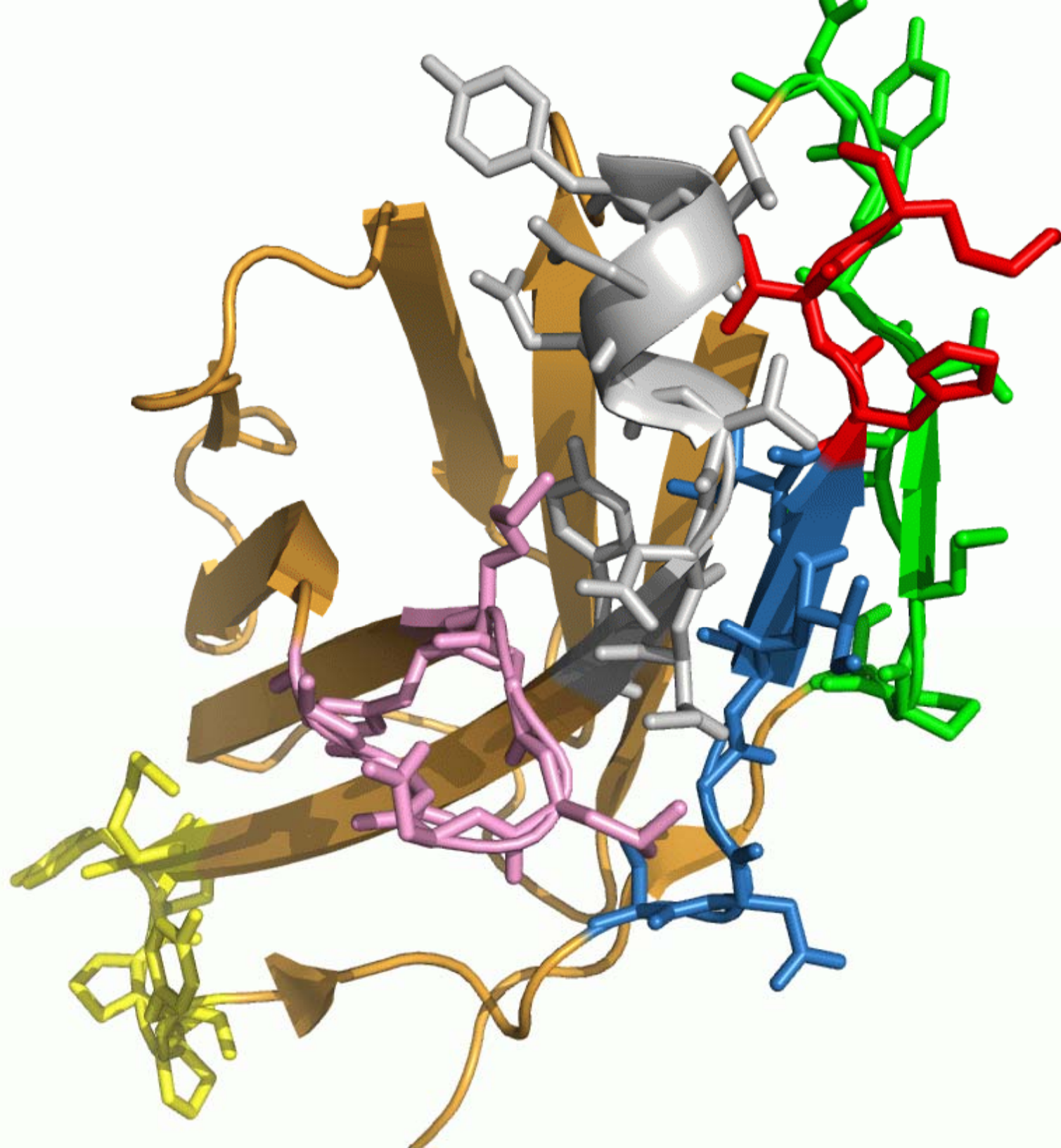

Supplement: Figure S4 — Antagonist peptides in the CD28 dimer interface. A still of Movie S1. In CD28 extracellular domain (1yjd.pdb), location is shown of homodimer interface peptides p1TA (HVK resolved in 1yjd.pdb is red), p2TA (green), p3TA (pink), p4TA (grey), and p5TA (SNGTII is blue) (see Figure 4A); MYPPPY is yellow. (PDF) [file pbio.1001149.s004.pdf]

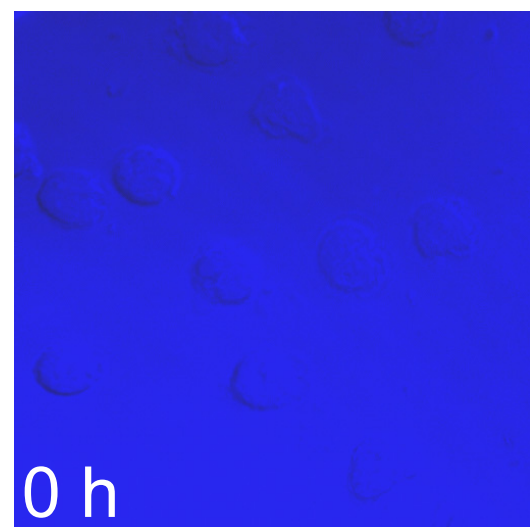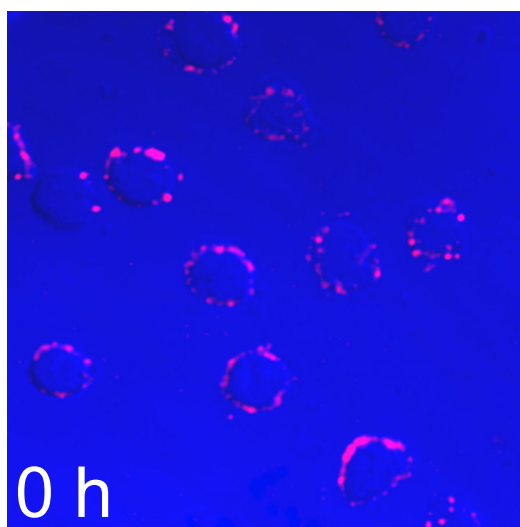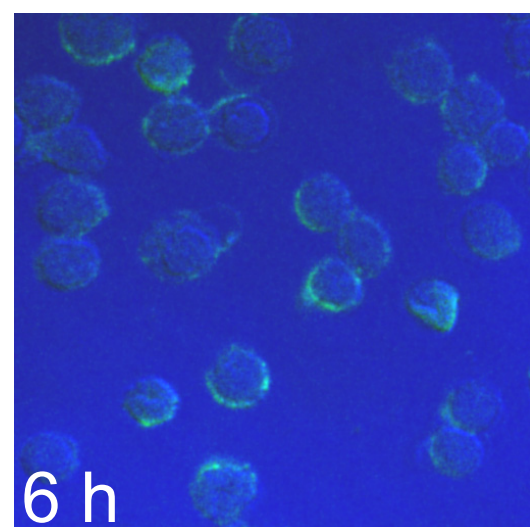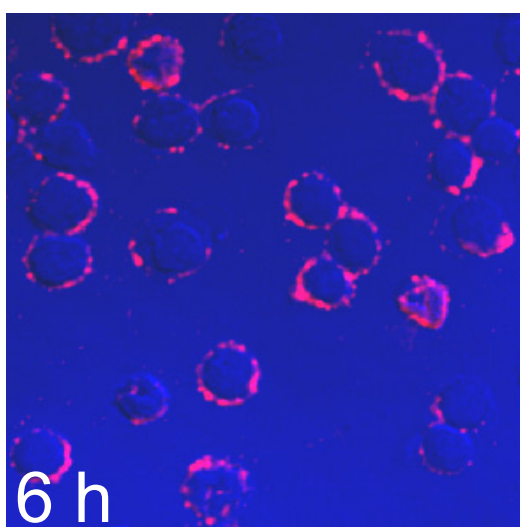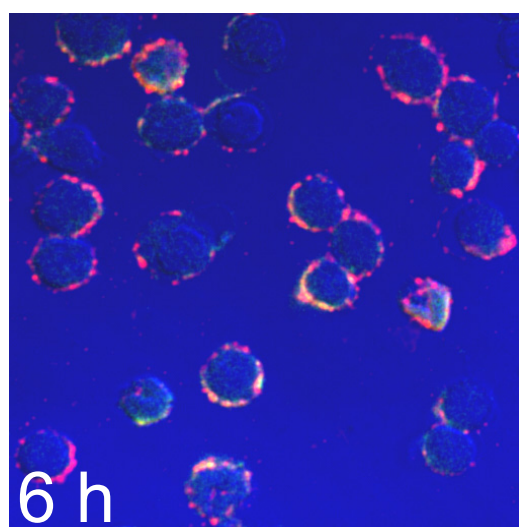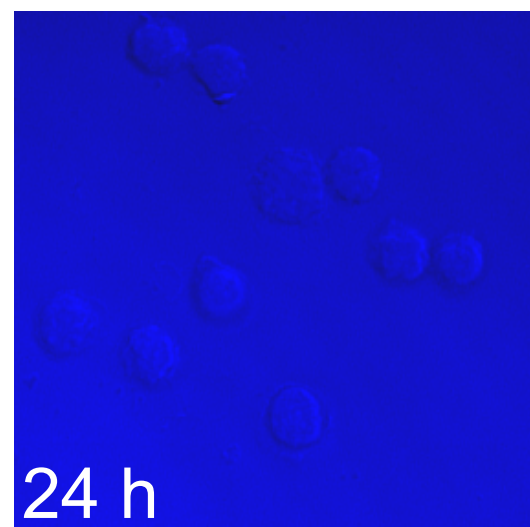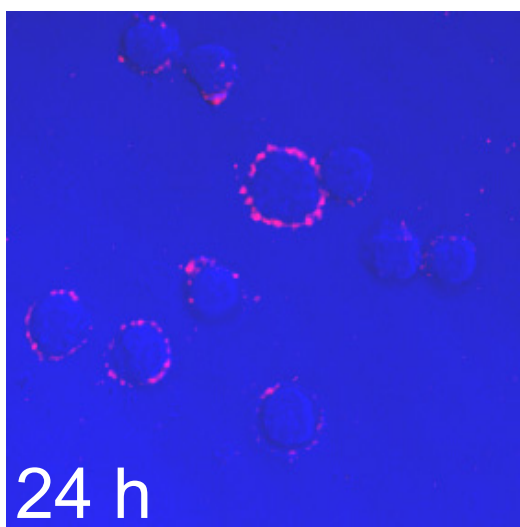

Merge

$\alpha$ CD28 mAb

CD28 Ab

Supplement: Figure S5 — SEB induces a transient change in surface accessibility of CD28 epitope HVKGKHLCP. CD4 cells were enriched to 90% from PBMC by use of RosetteSep (Stem Cell Technologies), incubated at a density of 4×10^6^ cells/ml and before addition of SEB (0 h), or at times indicated, washed, and stained with αCD28 mAb (secondary Ab: Cy-2, green) or goat polyclonal CD28 Ab (secondary Ab: Cy-3, red). Confocal fluorescence microscopy is shown. Merge, double staining with both antibodies. (PDF) [file pbio.1001149.s005.pdf]
