# Supplementary material for: Binding of Superantigen Toxins into the CD28 Homodimer Interface Is Essential for Induction of Cytokine Genes That Mediate Lethal Shock
Source: PLoS Biol. 2011 Sep 13;9(9):e1001149. doi: 10.1371/journal.pbio.1001149 (PMC3172200; doi:10.1371/journal.pbio.1001149)
Supplement: Table S1 — Kinetic parameters of surface plasmon resonance analysis in Figure 5. (PDF) [file pbio.1001149.s007.pdf]

Table S1 Kinetic parameters of surface plasmon resonance analysis in Figure 5.

Panel	Ligand	Analyte	k_a (1/Ms)	SEM (k_a)	% SEM (k_a)/k_a	k_d (1/s)	SEM (k_d)	% SEM (k_d)/k_d	KD (μM)	χ^2
**b**	CD28	SEB	320	3.78	1.18	7.28E-04	9.57E-06	1.31	2.28	1.28
**c**	p*1TA*	SEB	1,680	51.10	3.04	8.19E-04	1.35E-05	1.65	0.49	0.22
**d**	p*2TA*	SEB	279	6.64	2.37	7.87E-04	1.18E-05	1.50	2.82	0.68
**f**	p*1TA*	SEA	7,250	427.00	5.89	3.68E-03	5.89E-05	1.60	0.51	0.92
**g**	p*2TA*	SEA	523	13.60	2.60	3.33E-04	4.50E-06	1.35	0.64	0.42

Purified recombinant superantigens were used. k_a, association rate; k_d, dissociation rate; KD, dissociation constant; Ms, millisecond; SEM, *n*=3.
