# Supplementary material for: Binding of Superantigen Toxins into the CD28 Homodimer Interface Is Essential for Induction of Cytokine Genes That Mediate Lethal Shock
Source: PLoS Biol. 2011 Sep 13;9(9):e1001149. doi: 10.1371/journal.pbio.1001149 (PMC3172200; doi:10.1371/journal.pbio.1001149)
Supplement: Table S2 — Kinetic parameters of surface plasmon resonance analysis in Figure 6. (PDF) [file pbio.1001149.s008.pdf]

Table S2 Kinetic parameters of surface plasmon resonance analysis in Figure 6.

Ligand	Analyte	k_a (1/Ms)	SEM (k_a)	% SEM (k_a)/k_a	k_d (1/s)	SEM (k_d)	% SEM (k_d)/k_d	KD (μM)	χ^2
CD28	*wt*	603	2.69	0.43	9.87E-04	2.37E-05	2.40	1.64	1.34
CD28	*tk2*	757,000	18,400	2.43	4.74E-03	8.47E-05	1.78	6.3E-03	1.32
p*1TA*	*tk2*	1,300,000	16,500	1.27	6.54E-04	3.41E-05	5.21	5.0E-04	0.49
p*2TA*	*tk2*	1,200,000	13,900	1.16	5.38E-04	8.2E-05	15.24	4.5E-04	1.91

Purified recombinant superantigens were used. k_a, association rate; k_d, dissociation rate; KD, dissociation constant; Ms, millisecond; SEM, *n*=3.
